# *Aedes aegypti* Mosquito Detection at Bus Stations, Bogota, Colombia, 2023–2024

**DOI:** 10.3201/eid3106.241052

**Published:** 2025-06

**Authors:** Andrea Villamizar Gomez, Sandra Patricia Pérez Español, Marco Alexander Figueroa Caicedo, Andrea Camila Márquez Nossa

**Affiliations:** Subred Integrada de Servicios de Salud Sur Occidente E.S.E., Bogota, Colombia (A. Villamizar Gomez, M.A. Figueroa Caicedo); Secretaría Distrital de Salud de Bogota, Bogota (S.P. Pérez Español, A.C. Márquez Nossa)

**Keywords:** Aedes aegypti, vector-borne infections, Bogota, Colombia, surveillance

## Abstract

We monitored mosquitoes in 3 bus stations in Bogota, Colombia, located at 2,625 m above sea level. During December 2023–January 2024, we collected 27 larvae and 1 adult female *Aedes aegypti* mosquitoes at 1 station. Detection of *Ae. aegypti* mosquitoes in Bogota is a call to continue monitoring mosquitoes at stations.

*Aedes* spp. mosquitoes can feed on many species, including humans (*1*,*2*). *Ae. aegypti* mosquitoes are a public health concern because they can transmit pathogens that cause some of the most common arboviral diseases, such as dengue fever, Zika, chikungunya, and yellow fever (*2*–*4*). Among the *Aedes* mosquito species, *Ae. aegypti* is the most widely studied because of its broad distribution range and widespread association with arboviral transmission, especially dengue virus (*2*,*4*). *Ae. aegypti* mosquitoes are found in tropical climates where temperatures range from 15°C to 30°C, and the altitude is generally <1,700 meters above sea level (masl). Some countries, such as Mexico, Peru, and Bolivia, have reported *Ae. aegypti* mosquitoes at >2,000 masl (*1*,*4*–*6*). However, some reports in Colombia note *Ae. aegypti* mosquitoes at altitudes as high as 2,100 masl (*7*,*8*). *Ae. aegypti* mosquitoes are found in most urban and peri-urban areas of Colombia, according to a survey published by the National Health Institute (*7*).

In Colombia, dengue fever is the most common arbovirus disease. In 2024, the country registered 27,649 cases: 15,926 (57.6%) persons showed mild symptoms, 11,419 (41.3%) showed moderate symptoms, and 304 (1.1%) had severe symptoms (*8*). A group of 10 states, Valle del Cauca, Cali, Tolima, Huila, Santander, Norte de Santander, Antioquia, Bolívar, Cundinamarca, and Meta, had 21,392 (77.4%) of those cases. Cundinamarca, the state in which Bogota is located, had 867 reports. Only 36 cases of Zika virus infection were recorded in Colombia in 2024, 12 (33.3%) of which occurred in Cundinamarca. Furthermore, 15 cases of chikungunya virus were documented; of those, 1 (6.6%) case was reported in Cundinamarca in the area around Bogota (*8*). Of note, Bogota is the only place in Cundinamarca with no reports of arboviruses, but notifications have been made in most neighboring municipalities at lower altitudes (200–1,700 masl). Bogota is at 2,600 masl and is considered outside the distribution range of the vectors.

Climate change has increased global temperatures, leading to new arboviral outbreaks. Recent studies have shown that *Ae. aegypti* mosquitoes now inhabit areas that were once outside their distribution range (*1*,*2*,*5*,*6*,*8*). The temperature in Bogota has consistently risen since the 1990s. In the mid-1960s, the average temperature per year was 12.6°C. In 2022, the average temperature reached 13.8°C; the highest temperature recorded was 25.1°C (*9*). That temperature increase suggests that Bogota may no longer be outside the distribution range of *Aedes* spp. mosquitoes. Herein, we report detection of *Ae. aegypti* mosquitoes in the city of Bogota, Colombia.

The possibility of an expansion in the distribution range of *Ae. aegypti* mosquitoes created the need for weekly monitoring and sample collection by the Secretaría Distrital de Salud (https://www.saludcapital.gov.co) of Bogota beginning in May 2023. The sampling efforts focused on the 3 bus stations of the city that have heavy traffic to and from areas of *Ae. aegypti* mosquito endemicity.

We set up a total of 5 traps per bus station. We designed 3 traps to attract mosquitoes to lay eggs. We made the traps from dark plastic containers half filled with water; inside the containers, we put a flat wooden stick with rough surfaces designed to support the adhesion of eggs to the surface. We placed those traps in bathrooms and security guard booths. We also filled 2 larvae traps, made of discarded car tires containing water to allow egg development, and put them in green areas around each bus station. We also caught adults by using mosquito nets in the green areas and in surrounding buildings, such as bathrooms, offices, and security guard booths at each station.

We took all samples to the Secretaría Distrital de Salud for identification. During 1 year of surveillance, we identified 318 larval and 3,527 adult (1,862 females and 1,665 males) *Culex quinquefasciatus* mosquitoes, which is a species commonly reported in Bogota (*10*). In addition, in 1 bus station, during December 2023–January 2024, we caught 27 *Ae. aegypti* mosquito larvae in a larval trap and 1 adult female *Ae. aegypti* mosquito near the same trap; that species has never been reported in Bogota (*2*).

The bus stations are in urban areas that experience heavy travel of persons from other cities and towns in Colombia. Some of those urban areas are endemic for *Aedes* spp. mosquitoes (*7*). Mosquitoes might have arrived by bus and were likely caught in set traps or nets in an isolated event. However, those isolated events might lead to *Ae. aegypti* mosquito populations becoming established in Bogota. Therefore, we advise reinforcing hospital surveillance and notification systems to help identify local outbreaks of arbovirus infections. Detection of *Ae. aegypti* mosquitoes in Bogota is a call to set up a permanent monitoring program for mosquito species at bus stations in the city.
